# Gasless endoscopic thyroidectomy *via* modified areola approach with a simple flap-lifting technique

**DOI:** 10.3389/fendo.2022.1028805

**Published:** 2022-12-23

**Authors:** Tao Guo, Zehui Wu, Juntong He, Defeng Liu, Hong Wan, Yangyang Li, Shihao Peng, Aman Xu

**Affiliations:** ^1^ Department of General Surgery, The First Affiliated Hospital of Anhui Medical University, Hefei, Anhui, China; ^2^ Anhui Public Health Clinical Center, Hefei, Anhui, China

**Keywords:** endoscopic thyroidectomy, areola approach, flap lifting, CO2 insufflation, Kirschner wire, complication

## Abstract

**Objective:**

Studies have shown that carbon dioxide (CO2) insufflation during endoscopic thyroidectomy is associated with many risks. Recently, we have designed a simple lifting tool using Kirschner wire. We aimed to use this tool for flap-lifting in modified areola approach endoscopic thyroidectomy and compare it with conventional CO2 insufflation.

**Methods:**

In a prospective study, patients who underwent endoscopic thyroidectomy *via* modified areola approach were randomly assigned into gasless (n = 20) or CO2 groups (n = 22). Pre-operative variables included age, gender, tumor diameter, and clinical diagnosis. Intra-operative hemodynamic monitoring included mean arterial pressure, heart rate, pulse oximetry, end-tidal carbon dioxide (ET-CO2) and arterial pH. Other intra-operative details included total operative time, operative blood loss, conversion from endoscopic surgery to open surgery, intra-operative events, and endoscope video score. Postoperatively, the hospital stay, drainage volume, and complications were recoded.

**Results:**

Patient characteristics were not different between the two groups. During the operation, ET-CO2 levels were significantly higher in the CO2 group (*P* < 0.05), whereas arterial pH levels were significantly lower (*P* < 0.05). The CO2 group had longer operation time and higher endoscope clarity VAS score than gasless group. Hospital stay, drainage volume, and postoperative complications did not differ significantly between the two groups (*P* > 0.05).

**Conclusions:**

The gasless endoscopic thyroidectomy we performed *via* our Kirschner wire hook was safe, feasible, and yielded good results.

## Introduction

Because of its good cosmetic effect, scarless (in the neck) endoscopic thyroidectomy (SET) has developed rapidly in the past two decades (1). A number of innovative surgical approaches have been proposed, including the axillary approach, anterior chest approach, areolar approach, transoral approach, retroauricular approach, and various combinations of these approaches.

However, successful endoscopic thyroidectomy also depends on sufficient operating space, which is a necessary condition for clear vision. Currently, there are two main methods to maintain the operating space: carbon dioxide (CO2) insufflation and flap-lifting technique (gasless). Insufflation with CO2 is more prevalent, but it carries many risks, including hypercarbia, acidosis, pneumomediastinum, pneumothorax, subcutaneous emphysema, and cardiac arrhythmias (2–6). Animal experiments have shown that prolonged CO2 insufflation at high pressure will decrease cervical venous blood flow, and increase intracranial pressure and cerebral edema during endoscopic thyroidectomy (7, 8). The gasless technique, however, eliminates all these gas-related complications, enabling more patients to tolerate SET. Besides, it reduces the cost of CO2 insufflation as well as intensive end-tidal carbon dioxide (ET-CO2) and arterial blood gas monitoring during operation. At present, the gasless technique is widely used in SET with the transaxillary approach. While in other SET approaches, the gasless technique is less used, possibly due to a lack of suitable commercial suspension equipment.

Kirschner wire (K-wire) is widely used for the fixation of fractures and dislocations due to its easy accessibility, reliability, and affordability. Recently, we have designed a simple lifting tool for SET using K-wire. The purpose of this study is to investigate the safety, feasibility, and postoperative outcomes of gasless endoscopic thyroidectomy *via* a modified areola approach with this lifting tool.

## Patients and methods

### Patients

Between August 2021 and May 2022, a total of 42 patients with thyroid nodules underwent endoscopic thyroidectomy at The First Affiliated Hospital of Anhui Medical University (North District). Inclusion criteria (1): patient having a strong desire for cosmesis (2); benign tumor less than 4 cm in diameter, or differentiated thyroid carcinomas that is smaller than 2 cm in diameter and do not invade adjacent organs (3); no lateral cervical lymph node or distant metastasis was detected before the operation. Exclusion criteria (1): previous history of thyroid surgery (2); patients who cannot tolerate general anesthesia or long-time operation (3); medullary thyroid carcinoma or undifferentiated thyroid carcinoma.

All patients routinely underwent clinical examination, ultrasonography, fine needle aspiration cytology, laryngoscopy and vocal cord examination, serum thyroid profile tests, including T3, T4 and thyroid-stimulating hormone (TSH) levels. Whenever a malignant nodule was suspected or diagnosed by preoperative pathology, contrast-enhanced computed tomography (CT) scans of the neck were recommended. All patients provided informed written consent, and ethical approval was obtained from the Research Ethics Committee of the University of Anhui Medical University (North District), Anhui Public Health Clinical Center (2021-LL-05). The endoscopic thyroidectomies were all performed by one surgeon. During the operation, the patients were randomly assigned to the use of either CO2 insufflation or flap-lifting technique.

Pre-operative variables included age, gender, tumor diameter, and clinical diagnosis. The clinical diagnosis was obtained by comprehensive evaluation of fine needle aspiration cytology, sonography and CT results. Intra-operative hemodynamic monitoring included mean arterial pressure, heart rate, pulse oximetry, ET-CO2 and arterial pH. All these parameters were recorded at the following time points: before operation (T0), 60 min after trocar placement (T1), and at the end of surgery (T2). Other intra-operative details included total operative time, operative blood loss, conversion from endoscopic surgery to open surgery, intra-operative events, and endoscope video clarity score. The endoscope video clarity score was evaluated with a visual analog scale (VAS) which consists of a 10-cm line with anchor statements on the left (worst visual field) and on the right (excellent visual field) (9). Another doctor graded the score on the VAS based on intraoperative image definition, smoke effects on laparoscopic visibility, and endoscope cleaning times during the procedures. The hospital stay, drainage volume, as well as postoperative complications were recoded, including subcutaneous emphysema, pneumomediastinum, pneumothorax, hypocalcemia, transient recurrent laryngeal nerve (RLN) palsy, permanent RLN palsy, hemorrhage, skin ecchymosis, subcutaneous fluid retention, and wound infection.

### Surgical procedures

The surgical steps are similar to the procedure described by Wang (10). Under general anesthesia with endotracheal intubation, the patient is placed in the supine position with a shoulder pillow placed behind the neck, so the neck can be slightly extended. The surgeon stands between the two legs of the patient. The camera assistant stands to the right of the patient, while the scrub nurse stands to the left. The second assistant stands by the patient’s neck.

**Figure 1:**
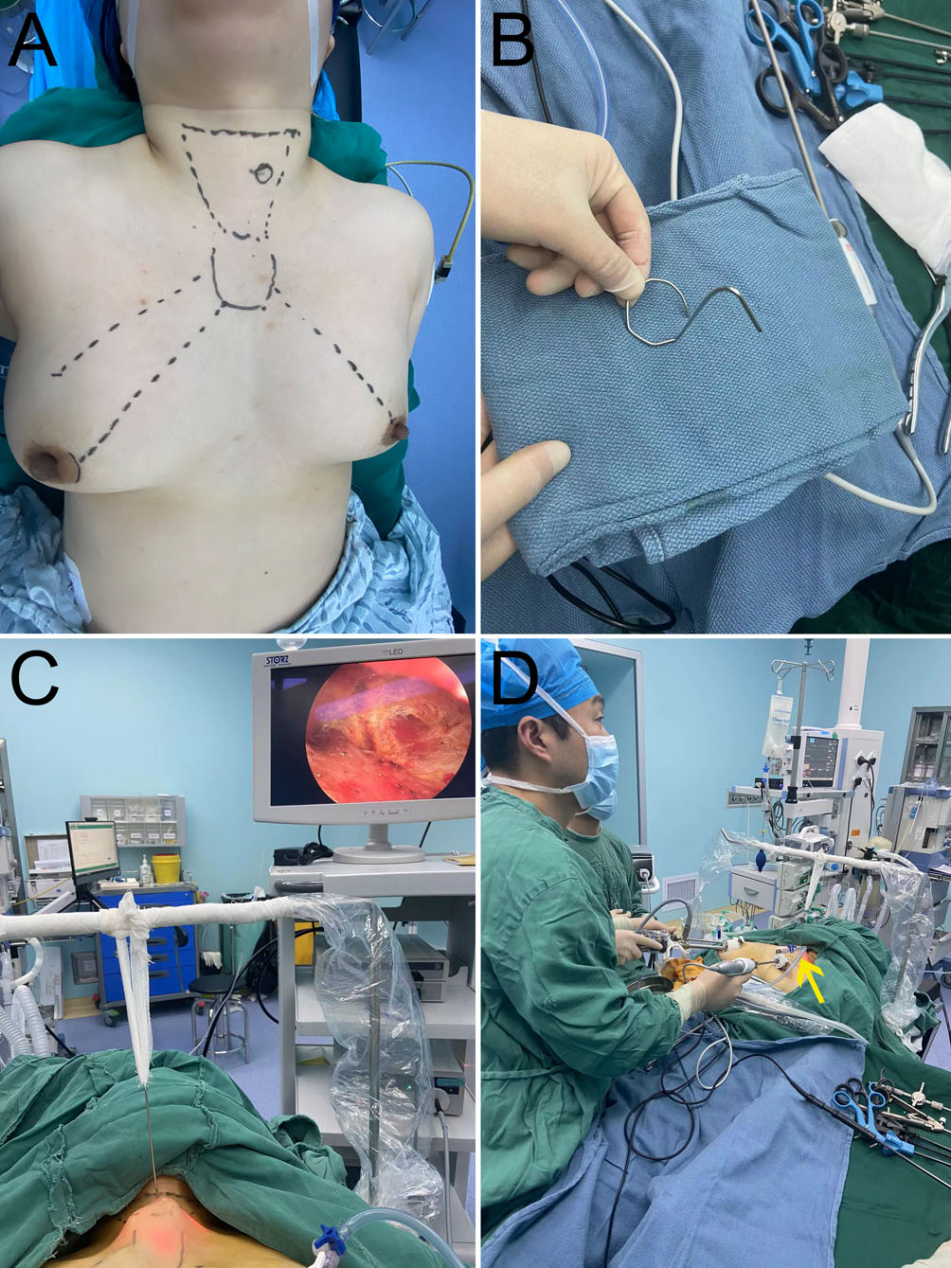
**(A)** Operation area and incision design; **(B)** Appearance of K-wire hook; **(C)** External schematic diagram of K-wire hook suspension device; **(D)** Overall surgical scene (yellow arrowheads: smoke suction tube).

As shown in **Figure 1A**, the operation area and incision design are marked preoperatively. From 2 o’clock to 4 o’clock position of the right areola border, a 12-mm curved incision is made as an observation hole. Through it, about 20 mL of diluted epinephrine solutions (prepared with 1 mL adrenaline in 500 mL normal saline) are injected subcutaneously to create an anatomical plane and reduce intraoperative bleeding. A tunnel is then made by bluntly dissecting the subcutaneous tissue with ovary forceps. Then, a 12-mm trocar is placed and a 30-degree face-down endoscope is inserted for observation. Under direct vision, a 5-mm trocar is placed at the 11 o’clock position of the left areola border as the first operation hole. To not interfere with the observation hole, we position another 5-mm trocar at 3 cm above the right nipple as the second operation hole. Because this kind of trocar placement is slightly different from the conventional complete areola approach in that all three incisions are close to the areola (10), we name our method the modified areola approach. Then, grasping forceps and an electric hook (or harmonic scalpel) are inserted to sharply separate the cord-like connective tissue in the cavity and enlarge the subcutaneous operation space of the anterior chest wall. Next, the bilateral sternocleidomastoid muscles are used as anatomical markers to dissect the subplatysmal space. At this time, for the CO2 group, we insufflate CO2 at the pressure of 6–8 mmHg to maintain the working space, while for the gasless group, we place the self-made hook to lift the flap. **Figure 1B** shows the appearance of the K-wire hook. The hook is made based on the anticipated size of the operation space during the operation. Fold the K-wire as needed, then form one end into an “O” shape and the other end into an inverted “J” shape. At the level of the suprasternal fossa, a 1-mm incision is made using #11 surgical blade at the anterior cervical flap. Then, the “O” shape end of the hook is inserted through the incision. Under the monitor of endoscopy, slowly insert the entire “O” shape ring along its arc into the deep surface of the flap. After that, the cervical flap is lifted vertically upwards. The inverted “J” shape end of the hook is connected to reversed L-shaped bar, which is fixed to the operating table near the patient’s head. The height of the bar can be adjusted until the flap is just tight (**Figure 1C**). To minimize the smoke generated when the energy equipment works, for both groups we connected a suction device to the trocar (**Figure 1D**).

By using the harmonic scalpel, the anatomical plane is extended from caudal to cephalad. The space scope is as follows: the lower boundary reached the superior sternum fossa, the upper boundary reached the level of the thyroid cartilage, and both sides reached the sternocleidomastoid muscle.

The strap muscles are dissected along the midline and retracted laterally for exposure of the thyroid gland. Longitudinally cut the isthmus and dissect upward along the gap between thyroid gland and trachea until the identification of the lateral branch of the superior laryngeal nerve. The superior artery of thyroid gland and its surrounding veins are coagulated as close as possible to the gland to ensure the blood supply to the superior parathyroid glands. Then turn to the inferior pole of the thyroid gland, expose and protect the inferior parathyroid glands. Tracheoesophageal groove areas are bluntly separated to expose the RLN. Upon confirming the path of the RLN toward its entry point into the larynx, dissect the thyroid lobe. The specimens are taken out through the central trocar and sent to frozen section. If it is malignant, lymph nodes of the central neck compartment are dissected. A drainage tube is routinely placed at the end of the surgery.

### Postoperative management

The patients were carefully checked for signs of bleeding, RLN injury, or difficulty breathing. RLN injury is defined as impaired movement of the vocal cords on laryngoscopy postoperatively. On the day of surgery, a liquid diet was provided. Then, soft oral foods were permitted on the second day after operation. Postoperative oral antibiotics were prescribed for 3 days.

### Statistical analysis

Statistical analysis is performed with SPSS 26.0 software (SPSS Inc., Chicago, IL, USA). Continuous data are presented as mean ± standard deviation (SD) or median (range), and categorical data are presented as number (%). Student’s t-test or Mann–Whitney U test is used for comparing continuous data, and the chi-square test or Fisher’s exact test is adopted for comparing categorical data. Pearson’s correlation method is used to assess the relationship between two continuous variables. *P* < 0.05 is considered statistically significant.

## Results

### Patient baseline characteristics

A total of 42 patients with thyroid nodules underwent modified areola approach endoscopic thyroidectomy during this period. They were randomly assigned to the CO2 group (n=22) and the gasless group (n=20). The preoperative demographics and clinical characteristics are shown in **Table 1**. Both groups were comparable in terms of age and gender (*P* > 0.05, **Table 1**). There was no significant difference between the two groups in terms of tumor diameter and preoperative clinicopathological diagnosis (*P* > 0.05).

**Table 1:** Demographics and clinical characteristics of the two groups.

	CO2 group (n = 22)	Gasless group (n = 20)	*P*
Mean age (years)	31.1 ± 5.6	32.0 ± 8.5	0.680
Gender			1.000
Female (%)	20 (95.5%)	19 (95.0%)	
Tumor diameter (cm, average diameter)	1.0 ± 0.3	1.0 ± 0.2	0.494
Pre-operative clinicopathological diagnosis			0.753
Benign	4	2	
Malignant	18	18	

### Intraoperative variables

All patients underwent endoscopic thyroidectomy successfully. There was no conversion to open surgery in both groups. Hemodynamic parameters were measured before surgery (T0), 60 minutes after trocar placement (T1), and at the end of surgery (T2) (**Table 2**). At all these time points, mean arterial pressure, heart rate, and pulse oximetry were not different between the two groups (*P* > 0.05). There was no significant difference between the two groups in ET-CO2 and arterial pH before surgery (*P* > 0.05). However, after 60 minutes of trocar placement, ET-CO2 levels were significantly higher in the CO2 group than in the gasless group (*P* < 0.05), whereas arterial pH levels were significantly lower (*P* < 0.05). This kind of difference still existed at the end of the operation (*P* < 0.05).

**Table 2:** Intra-operative hemodynamic parameters of the two groups.

	CO2 group (n = 22)			Gasless group (n = 20)			*P*		
	T_0_	T_1_	T_2_	T_0_	T_1_	T_2_	*P_1_*	*P_2_*	*P_3_*
Mean arterial pressure (mmHg)	73	78	79	74	77	77	0.240	0.612	0.085
Heart rate (beats/min)	70	73	74	70	71	71	1.000	0.213	0.071
Pulse oximetry	99%	99%	99%	99%	99%	99%	0.977	0.763	0.982
ET-CO2 (mmHg)	32	40	40	33	35	35	0.379	<0.001	<0.001
Arterial pH	7.44	7.38	7.35	7.43	7.42	7.41	0.509	<0.001	<0.001

T0: before surgery.

T1: at 60 min after trocar placement.

T2: at the end of surgery.

P_1_, P_2_, P_3_: p value for difference between the groups at T0,T1 and T2, respectively.

ET-CO2: End-tidal carbon dioxide.

As shown in **Table 3**, the CO2 group had a longer total operation time than the gassless group, and its endoscope clarity VAS score was lower than that of the gassless group. In terms of operative blood loss, conversion to open surgery, intra-operative events, and RLN not identified event, there was no significant difference between the two groups.

**Table 3:** Intraoperative variables of the two groups.

	CO2 group (n = 22)	Gasless group (n = 20)	*P*
Total operative time (min)	135	115	<0.001
Operative blood loss (ml)	60	55	0.051
Conversion	0	0	–
Intra-operative events	0	0	–
RLN not identified	0	0	–
Endoscope clarity VAS	6.8	8.1	<0.001

RLN, recurrent laryngeal nerve.

VAS, endoscope visual analogue score.

### Post-operative outcomes

**Figure 2:**
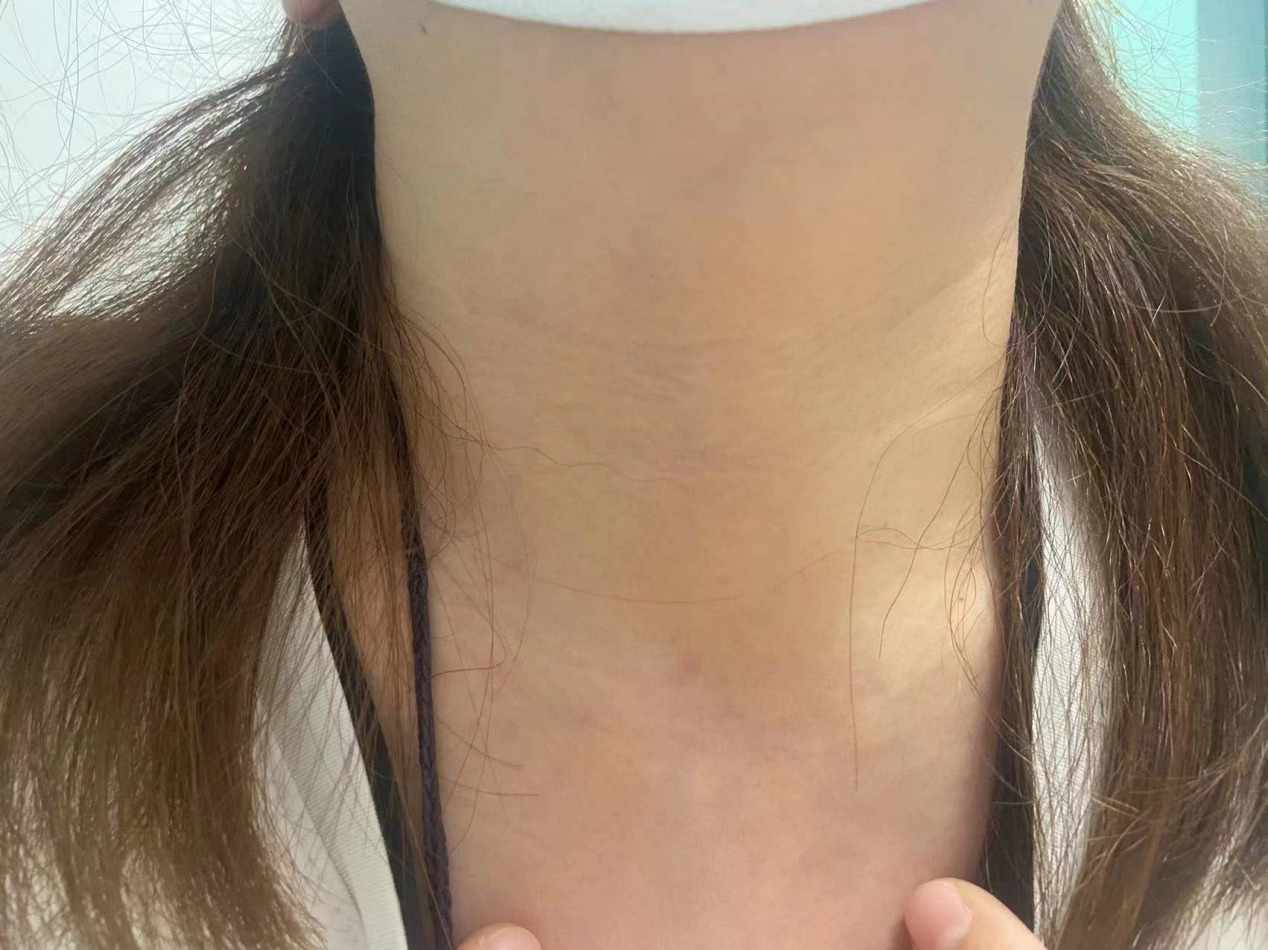
Three months after surgery, an anterior neck image of a patient in the gasless group.

There was no significant difference between the two groups in hospital stay, drainage volume and postoperative complications (*P* > 0.05, **Table 4**). Neither transient or permanent RLN palsy was found postoperatively in both groups. However, there was one case of subcutaneous emphysema and one case of pneumomediastinum among the CO2 group. Patients in the gasless group do not display significant surgical scar on the anterior neck during postoperative follow-up (**Figure 2**).

**Table 4:** Post-operative outcomes of the two groups.

	CO2 group (n = 22)	Gasless group (n = 20)	*P*
Hospital stay (days)	4.5	4.6	0.729
Drainage volume (ml)	20	19.5	0.536
Postoperative complications	0	0	–
Subcutaneous emphysema	1	0	–
Pneumomediastinum	1	0	–
Pneumothorax	0	0	–
Hypocalcemia	0	0	–
Transient RLN palsy	1	1	1.000
Permanent RLN palsy	0	0	–
Hemorrhage	0	0	–
Skin ecchymosis	0	0	–
Subcutaneous fluid	0	0	–
Wound infection	0	0	–
Tracheal injury	0	0	–
Esophagus injury	0	0	–

RLN, recurrent laryngeal nerve.

## Discussion

Thyroid tumors are the most common malignant tumors of the endocrine system and occur more often in females. The main treatment for thyroid tumors is surgical resection. Traditional open thyroidectomy (OT), considered the gold standard, is widely performed around the world. However, OT will inevitably leave a visible scar on the anterior neck, which negatively affects body image or even the quality of life (11, 12). Endoscopic surgery was first described by Gagner et al. in 1996 for secondary hyperparathyroidism. Then, in 1997, Yeung in Hong Kong and Hüscher in Italy published the first reports on endoscopic thyroid surgery (13). After two decades of development, endoscopic thyroidectomy has become more and more perfect and popularized, and now a variety of surgical methods have become available.

Creating and maintaining an operating space is the first step in endoscopic thyroidectomy, which is critical to the smooth implementation of follow-up procedures. CO2 insufflation is a widely used method to maintain the operating space. However, CO2 is highly soluble in blood and diffuses easily in loose areolar and rough tissue, such as in the neck (14). During endoscopic thyroidectomy surgery, special attention should be paid to CO2 insufflation pressure. Otherwise, high CO2 pressure or excessive absorption of CO2 may adversely affect patients’ respiratory, circulatory and other systems and increase the possibility of related complications. It is worth noting that even if CO2 insufflation pressure is < 8 mmHg, CO2 embolism can still occur (15).

In contrast, the gasless technique eliminates all the CO2-related complications, enabling more patients to tolerate edoscopic thyroidectomy. In 1998, Shimizu horizontally inserted two pieces of K-wires into the subcutaneous tissue in front of the neck and connected them to an abdominal wall lifting device (16). The method obtained a good operation field of vision for video-assisted neck surgery (16–18). For scarless endoscopic thyroidectomy, gasless techniques are mostly used in the axillary approach. When it comes to the bilateral areolar approach that can provide good access to bilateral thyroid lobes, gasless techniques are rarely reported. This may be due to the lack of suitable commercial lifting equipment.

In this study, we devised a simple flap-lifting tool using a piece of K-wire. Using this tool, we performed gasless bilateral areolar approach endoscopic thyroidectomy. Our gasless group do not require CO2 insuffulation throughout the entire operation process. The results showed that the vital signs were stable and arterial pH were within the physiologic range during surgery in both groups. However, following CO2 insufflation, ET-CO2 levels were significantly higher in the CO2 group than in the gasless group, whereas arterial pH levels were significantly lower in the CO2 group. In addition, the CO2 group had a longer total operation time than the gasless group. Smoke and vapor plumes generated by energy devices easily interfere with the operation field of view. Consequently, surgeons have to suction and clear endoscopes frequently, which affects operating cavity pressure and interferes with the procedure. In contrast, the gasless technique allows surgeons to do unlimited suction for bleeding or smoke clearance without loss of exposure. This explains the longer operation time and higher endoscope clarity VAS score in the CO2 group.

There was a slight increase in intraoperative blood loss in the CO2 insufflation group, but the difference is not statistically significant (P>0.05). This is likely due to the relatively small thyroid lesions in most of the study patients, which decreases the difficulty of surgery. In our study, all these operations were performed by one surgeon who had overcome the learning curve and was proficient in the treatment of perithyroid blood vessels. As shown in **Table 3**, the mean operation time for both groups was about 2 hours. Thus, there is no significant difference in operative blood loss between the two groups.

We used orthopedic surgical pliers for twisting and bending K-wire to make the lifting hook. In our experience, the curvature of the “O” shape ring must be smooth to facilitate the placement of the hook. To reduce the risk of damaging other tissues, we used endoscopy to monitor the placement of the lifting hook, which we consider to be extremely important. Collaboration with the manufacturer to improve and commercialize the device may reduce surgical risk and save operation time.

It would be fitting to acknowledge the limitations of our study. First, the sample size of our study is relatively small, which decreases the statistical power. Large sample size is needed to get a more reliable conclusion. Second, most cases in our study were microcarcinomas of the thyroid gland, therefore the results may not apply to all thyroid disorders. We hope to include more patients with other thyroid disorders in the future. Third, compared with open thyroidectomy, endoscopic thyroidectomy has a longer learning curve. As a result, the success of endoscopic thyroid surgery is greatly influenced by the experience of the operators. In our study, we relied on the experience of two surgeons (GT and HJ) rather than many surgeons with different levels, therefore the result may be biased.

## Conclusion

Our study showed that gasless endoscopic thyroidectomy *via* a modified areola approach with this lifting tool is safe, feasible, and has good postoperative results. Moreover, K-wire is cheap and easy to find. Consequently, our method is easy to popularize, reducing the difficulty of endoscopic thyroid surgery.

## Data availability statement

The raw data supporting the conclusions of this article will be made available by the authors, without undue reservation.

## Ethics statement

The studies involving human participants were reviewed and approved by The First Affiliated Hospital of Anhui Medical University (North District). The patients/participants provided their written informed consent to participate in this study. Written informed consent was obtained from the individual(s) for the publication of any identifiable images or data included in this article.

## Author contributions

TG and AX conceived the study. ZW, JH and DL collected the data. TG and ZW analyzed the data. HW, YL and SP provided guidance on experimental design, data analysis, and presentation of results. TG wrote the paper. ZW and AX revised the paper. All authors contributed to the article and approved the submitted version.
